# Effects of single‐ and double‐shift work on hand and cognitive functions in nurses

**DOI:** 10.1111/inr.13057

**Published:** 2024-11-06

**Authors:** Fadime Ulupinar, Sibel Meler

**Affiliations:** ^1^ Department of Nursing Faculty of Health Sciences, Erzurum Technical University Erzurum Turkey; ^2^ Faculty of Medicine Hospital Selçuk University Konya Turkey

**Keywords:** attention, hand function, response time, shift work, working memory

## Abstract

**Aim:**

This study aims to examine the influence of single and double‐shift work schedules on hand function and cognitive capacities, specifically working memory, attention, and response time in nurses.

**Background:**

Shift work, particularly in extended formats, is known to affect various physical and cognitive functions critical to nursing duties. Understanding these impacts is vital for managing nurse schedules to minimize health risks and maximize performance.

**Introduction:**

With an increase in demands on healthcare systems, nurses often endure prolonged working hours, which may impair their cognitive and manual abilities, thereby affecting patient care quality.

**Methods:**

This study involved 45 nurses aged 20–40 years from shift‐oriented units providing direct patient care. Hand function was assessed using the Nine‐Hole Peg Test (9‐HPT). Cognitive functions such as working memory, attention, and response time were evaluated using the digit span task and the Stroop test, respectively. Two‐way analysis of variance (ANOVA) was used for statistical analysis, assessing the interactions of time and shift type, with a significance level set at *p* < 0.05.

**Results or findings:**

The results revealed significant changes in all tested variables between pre‐ and post‐shift assessments and between single and double‐shift conditions. Notably, the double‐shift work significantly exacerbated declines in all measured functions.

**Discussion:**

These findings suggest that double shifts may intensify the deterioration of essential nursing skills, potentially compromising patient care.

**Conclusion and implications for nursing and/or health policy:**

This study underscores the detrimental effects of extended shift work on nurses' cognitive and manual functions. These insights should guide healthcare institutions in revising nurse scheduling practices to safeguard their well‐being and maintain high standards of patient care.

## INTRODUCTION

Across numerous nations, healthcare practitioners represent the dominant demographic within the shift‐working population, with the nursing profession forming the most significant portion of this healthcare workforce (Dall'Ora et al., 2016; Ganesan et al., 2019; González‐Caballero, 2024). To ensure the sustained 24‐h provision of health services to critically ill patients, this frontline cohort adopts unconventional working schedules, deviating markedly from the standard diurnal working paradigm (Devore et al., 2013; Merchaoui et al., 2017; Molzof et al., 2019). Despite the essential nature of this operational approach, it poses noteworthy challenges to the healthcare infrastructure, particularly due to its potential adverse implications on patient care (Dall'Ora et al., 2016; Ganesan et al., 2019; Killgore & Weber, 2014). The impact of the irregular circadian rhythm considerably undermines alertness and performance efficiency during on‐duty periods (Okechukwu et al., 2022; Topal Kılıncarslan et al., 2024; Weng & Chang, 2024). Various duty schedules necessitating early commencement or late terminations may further augment sleep deprivation and disruptions in the sleep‐wake cycle (Boivin & Boudreau, 2014; Chang et al., 2024; Molzof et al., 2019). Circadian rhythms, regulated by the suprachiasmatic nucleus in the hypothalamus, orchestrate a wide range of bodily functions, including sleep‐wake cycles, hormone release, and metabolism (Boivin & Boudreau, 2014; Killgore & Weber, 2014; Weng & Chang, 2024). Alterations in these rhythms due to shift work can lead to misalignment between the internal circadian clock and external environment, resulting in circadian rhythm sleep disorders. This misalignment affects the secretion of key hormones such as melatonin, which regulates sleep, and cortisol, which is involved in stress response and energy regulation (Bani Issa et al., 2020; Razavi et al., 2019). The consequent hormonal imbalance can impair cognitive functions, including attention, memory, and decision‐making processes, and can also influence motor skills, further exacerbating the risk of errors and accidents in healthcare settings (Ganesan et al., 2019; Wagstaff & Lie, 2011).

The International Council of Nurses recognized the indispensability of shift work within the nursing field but concurrently voiced significant concern over the detrimental consequences this could have on nurses' physical and mental health, as well as their capacity to offer superior patient care (International Council of Nursing, 2023). Forecasts on a global scale suggest a looming deficiency in the nursing workforce in the forthcoming years, accentuating the criticality of comprehending the elements that influence nurse retention (Auerbach & Staiger, 2017; BAE, 2024; Hassmiller, 2021). The nursing profession is universally recognized as stress‐intensive, with a multitude of studies investigating the psychological burden it may impose (Pulido‐Martos et al., 2012; Singh et al., 2020). Chronic exposure to stress, particularly in high‐stakes environments such as healthcare, can lead to significant alterations in neuro‐muscular function and cognitive processing abilities. Stress activates the body's fight or flight response, releasing cortisol and other stress hormones, which can affect brain function, including memory, attention, and decision‐making processes. Over time, this heightened state of alertness can impair neuro‐muscular coordination and cognitive performance, increasing the risk of errors in patient care (Ganesan et al., 2019; Killgore & Weber, 2014; Liu et al., 2023). Additionally, the physical demands of shift work, combined with stress‐induced muscle tension, can lead to musculoskeletal disorders, further impacting nurses' ability to perform their duties effectively (Caruso, 2014; Molzof et al., 2019). However, there remains a paucity of research specifically targeting the understanding of shift work's impact on precision neuro‐muscular functions and cognitive functions within the nursing purview (AL‐Hammouri et al., 2024; Devore et al., 2013; Ganesan et al., 2019). This gap in the literature underscores the need for comprehensive studies that explore the multifaceted effects of shift work on nurses, not only from a psychological perspective but also in terms of its physiological impacts on neuro‐muscular and cognitive functions.

Although isolated findings suggested that shift work did not debilitate short‐term tasks demanding concentration and working memory, the preponderance of empirical studies substantiates the strong association between long work hours and adverse outcomes for nurses (Wagstaff & Lie, 2011). One study corroborated that sleep deprivation, a common occurrence in shift workers, could deleteriously affect attention, with cognitive functionality potentially compromised even following a single night shift (Shao et al., 2010). Consistent with these findings, recent research indicated that circadian disruption and sleep disturbances pervasive among shift workers might detrimentally impact memory, learning, and retrieval of learned behaviors. Moreover, a separate study suggested that cognitive function could require a minimum of 5 years to return to baseline levels following cessation of shift work (Titova et al., 2016). The cumulative effects of disrupted sleep patterns and circadian misalignment on cognitive performance are profound. Prolonged exposure to such conditions can lead to significant deficits in executive functions, which are critical for planning, problem‐solving, and multitasking—skills essential for nursing. These cognitive impairments can manifest as a reduced ability to process complex information, make critical decisions, and maintain attention to detail, thereby increasing the likelihood of errors in patient care. Furthermore, the impact on memory can affect a nurse's ability to recall patient histories, medication dosages, and treatment protocols accurately, compromising patient safety and care quality. The interplay between sleep, circadian rhythms, and cognitive performance underscores the necessity of addressing shift work's challenges to safeguard nurses' cognitive health and, by extension, patient well‐being.

It is not uncommon for nurses to work overtime, often entailing extended work periods both daily and weekly (Bae & Fabry, 2014; Books et al., 2020; Kunaviktikul et al., 2015). Owing to the necessity of continuous nursing care, the standard weekday, daylight‐hours work schedule proves insufficient in meeting the 24‐h coverage requirement (Caruso, 2014; Kunaviktikul et al., 2015). To address this issue, many sectors have adopted work schedules that divide the day into either three 8‐h shifts or two 12‐h shifts (Wagstaff & Lie, 2011). In contrast, nurses in Turkey operate under a different model (Ministry of Health of the Republic of Turkey, 2023). Their schedule typically consists of an 8‐h day shift followed by a 16‐h evening‐night shift (Ministry of Health of the Republic of Turkey, 2023; Shao et al., 2010). After completing their shift, nurses are granted a minimum rest period of 24 h before they are scheduled to work again (Ministry of Health of the Republic of Turkey, 2023). Although providing sufficient rest periods for nurses, this working system also brings with it a long workload of 16 h, where sleeping is prohibited. The deterioration in the quality of nursing care due to disruptions in circadian rhythms and fatigue, especially in the latter part of the shift following a sleepless night, raises serious concerns (Devore et al., 2013; Esmaily et al., 2022; Hassmiller, 2021; Kunaviktikul et al., 2015). These negative impacts could have grave consequences for patient safety and the overall efficiency of healthcare delivery (Caruso, 2014; Devore et al., 2013; Kunaviktikul et al., 2015).

Recent prospective cohort studies have highlighted a general correlation between shift work and cognitive function deterioration, with extended shift durations potentially leading to more profound functional deficits (Devore et al., 2013; Ganesan et al., 2019; Molzof et al., 2019). This concern is particularly relevant in healthcare roles such as nursing, which require high levels of accuracy. The cognitive performance and vigilance of nurses are crucial for patient safety, as even minor lapses could have severe repercussions (Merchaoui et al., 2017; Titova et al., 2016). However, while the impact of shift work on health outcomes has been extensively studied, the specific effects of single versus double‐shift work patterns on nurses' cognitive and motor functions have not been adequately explored. The literature predominantly addresses the broader implications of shift work, leaving a gap in understanding how different shift structures—particularly the comparison between single and double shifts—affect nurses' hand function, working memory, attention, and response time. Addressing this gap, our study aims to investigate the distinct impacts of single‐ and double‐shift work on these critical functions. By doing so, we seek to provide a nuanced understanding of how shift work patterns contribute to cognitive and motor function alterations in nursing staff, thereby offering insights essential for improving nurse well‐being and patient care quality.

## METHODS

### Study design

**FIGURE 1:**
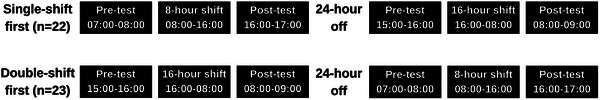
Study design and workflow processes (***Note***: Hand and cognitive functions were assessed during the tests).

This study, designed as a comparative descriptive study, was conducted in an academic medical institution under a non‐profit health system in Turkey from September 2023 to January 2024. The aim was to compare the effects of single and double‐shift work patterns on nurses' hand function, working memory, attention, and response time. The participants had a cyclical shift work arrangement, that is, a nurse concluding a single shift (08:00‐16:00) would proceed to a double shift (16:00‐08:00) subsequent to a 24‐h period of rest; conversely, a nurse concluding a double shift would embark on a single shift after a 24‐h recuperative interval (Figure 1). A comprehensive set of four assessments were administered to all participants, carried out both prior to and following a single shift, as well as before and after a double shift. In other words, a total of 180 test packages (one test package takes about 30 min) for 45 nurses were carried out, amounting to a total assessment time of 90 h. Hand function evaluations and cognitive testing were conducted individually for the participants within their designated rest areas, both before their shift's commencement and immediately upon its conclusion. 1 h prior to the start of a shift, i.e., between 07:00‐08:00 for a single shift and 15:00‐16:00 for a double shift, pre‐tests were carried out. Post‐tests were undertaken 1 h following the shift's termination, specifically 16:00‐17:00 for a single shift and 08:00‐09:00 for double shifts. Thus, the design of this study ensured an element of randomness and balancing in single‐ and double‐shift work schedules (Figure 1). All assessments were conducted by one researcher to ensure consistency, while a second researcher independently verified the results to maintain the highest standards of data integrity and reliability. This study obtained the necessary ethical approvals and permissions from the Erzurum Technical University Ethics Committee (Decision No: 5/9, dated August 17, 2023).

### Participants

The minimum sample size for this study was determined using the G*Power statistical software, specifically for an ANOVA: repeated measures, within factors analysis, with an alpha of 0.05, a power of 0.90, and considering a single group undergoing four measurements. This analysis indicated that a minimum of 30 participants would be sufficient to detect the expected effects. However, adopting a more conservative approach to account for potential data variability and participant dropout, we initially targeted enrolling 50 nurses. Ultimately, the study was completed with 45 nurses, ensuring the robustness of our findings despite the slight reduction in participant numbers.

Participants were systematically selected from a pool of nurses working in an academic medical institution under a non‐profit health system in Turkey. The process began with an initial screening to identify nurses who met the basic inclusion criteria: being registered nurses with at least 1 year of work experience, aged between 20 and 40 years, and having a healthy body mass index (BMI) within the standard range. This age range and BMI criteria were selected based on existing evidence, indicating that age and BMI significantly influence cognitive and physical performance (Pitrou et al., 2022; Salthouse, 2001; Xu et al., 2024). To minimize the variability introduced by age‐related cognitive decline, nurses aged 20–40 were included. Furthermore, a healthy BMI positively correlates with better cardiovascular health, which has been shown to support physical endurance and cognitive function (Pitrou et al., 2022; Xu et al., 2024). Elevated BMI, particularly in the overweight or obese range, has been associated with a higher risk of cognitive decline and reduced physical performance in later life. These considerations were pivotal in defining our inclusion criteria to ensure that the study's results accurately reflect the cognitive and physical capabilities of a relatively homogeneous group of participants.

Eligible nurses were then invited to participate through email invitations and informational sessions, where the study's objectives, procedures, and potential benefits were explained in detail. Interested nurses provided informed consent, affirming their voluntary participation. Further screening was conducted to ensure participants met all inclusion criteria and none of the exclusion criteria, which included non‐nursing health professionals, nursing personnel not involved in direct patient care, advanced practice nurses, individuals with a history of psychiatric illnesses, or those on specific medication regimes. Participants were also advised to maintain their normal dietary and caffeine consumption habits to ensure the study's results would be as applicable as possible to real‐world scenarios.

### Hand functions

To assess the hand function of the participants, a commercial plastic version of the Nine‐Hole Peg Test (9‐HPT) was employed, with task completion times logged in seconds. The 9‐HPT necessitates that participants sequentially insert and subsequently remove nine pegs from nine corresponding holes as swiftly as possible. In a recent study, the 9‐HPT showcased excellent intra‐rater reliability (ICCs: 0.91–0.94) and good‐to‐moderate test–retest reliability (ICCs: 0.89–0.61). Furthermore, kinematic indexes such as normalized jerk and mean velocity, which strongly correlated with 9‐HPT scores (*r*‐score: 0.8–0.3), validated the 9‐HPT ’s ability to assess manual dexterity effectively. These findings affirm the 9‐HPT's utility in our study for evaluating hand function, underlining its precision and reliability (Temporiti et al., 2023). The 9‐HPT apparatus comprises a molded dish adjoined to a 9‐hole peg board, with dimensions of 31 × 26 × 4 cm, and nine plastic pegs, each measuring 0.6 cm in diameter. The peg board was positioned at the midline of the participants' body. Participants were instructed to be seated on a chair commensurate with their height, ensuring that the table top was at mid‐chest level. They were then asked to insert and remove all nine pegs using their dominant hand, one at a time, in as short a time span as possible; no specific sequence of placement was mandated. The score was determined by the total time, in seconds, required to complete the task, with the timer commencing upon contact with the first peg and concluding with the return of the final peg to the dish. The minimum time recorded from two attempts for dominant each hand was adopted as the criterion measure of dexterity.

### Cognitive performance

The assessment of working memory was performed using the digit span task, a constituent of the Wechsler cognitive test, whereas attention and response time were measured using the Stroop test. In the Wechsler working memory test, a sequence of numbers, ranging from three to nine digits, is presented to the participant, who is then requested to repeat the series in either a forward or backward order. The test concludes after the participant makes two errors. This test is validated for its reliability and effectiveness in cognitive assessment, as evidenced by a meta‐analysis with effect sizes up to 1.34 (Jasinski et al., 2011).

The Stroop cognitive test is used to derive the interference score and response time, thus facilitating the appraisal of executive control, inhibitory control, and reaction time. The Stroop Color and Word Test's proven sensitivity and specificity in assessing executive function deficits validate its application in our study for cognitive evaluation (Homack & Riccio, 2004). To curtail test duration and eliminate potential learning effects, non‐essential preparatory stages were excluded from this study, with only the principal stage executed. Throughout the test, four words (green, blue, red, and yellow) are displayed on a monitor screen for a 2‐s period, separated by intervals of 0.008 s. The screen then shows 48 congruent word‐color pairs (e.g., “green” in green color) and 48 incongruent word‐color pairs (e.g., “green” in yellow color). The participant is expected to respond to the color of the stimuli while ignoring its semantic meaning, and press the respective key on the keyboard. Attention is determined by calculating the interference score, which is the difference between the correct congruent word‐color responses and accurate incongruent word‐color responses. Response time is computed from the interference time, indicative of the variance in response times to incongruent and congruent word‐color stimuli.

### Statistical analysis

Data processing and statistical analyses were performed using IBM SPSS Statistics v25 for Windows (IBM Corp, Armonk, NY, USA) and GraphPad Prism version 9.0 for Windows (GraphPad Software, La Jolla, CA, USA). All data were represented as means ± standard deviation (SD). To evaluate the interactions of time (pre‐ and post‐shift) and shift type (single and double), repeated measures of two‐way ANOVA were employed. The sphericity assumptions were verified using Mauchly's test, and corrections were implemented when an assumption was found to be violated. Specifically, if the epsilon (ε) value was less than 0.75, the Greenhouse–Geisser correction was applied, whereas the Huynh–Feldt correction was utilized for the degree of freedom when ε was greater than 0.75. *p* value less than 0.05 was considered indicative of statistical significance. Moreover, effect sizes for pre‐ and post‐shift changes were computed using Cohen's d (Cohen, 2013).

## RESULTS

The study encompassed a total of 45 nurses. The mean ± SD values for their respective ages, work experience, and BMI were calculated as 25.2 ± 4.7 years, 4.8 ± 4.2 years, and 21.9 ± 1.3 kg m^−2^.

Table 1 lists the results of the analysis examining whether the variables of hand function, working memory, interference score, and response time exhibit differences in both pre‐ and post‐shift states, as well as between single‐ and double‐shift works. The findings demonstrated significant results for all variables in both the before–after comparison and the single–double comparison. Furthermore, the analyses revealed the interaction of time (pre‐ vs. post‐) and shift type (single‐ vs. double‐) significance across all variables, indicating that a double shift induced greater change.

**TABLE 1:** Nurses’ hand functions and cognitive performance comparisons of time (pre‐ and post‐shift) and shift type (single‐ and double‐shift).

	Single‐shift work		Double‐shift work		Statistical analyses
	Pre‐shift	Post‐shift	Pre‐shift	Post‐shift	
Hand functions	18.5 ± 2.8	18.9 ± 2.6	18.6 ± 2.9	19.8 ± 3.0	Pre vs. post: *p* = 0.001 Single vs. double: *p* < 0.001 Time × shift type: *p* < 0.001
Working memory	7.5 ± 1.0	7.2 ± 0.8	7.5 ± 0.9	6.6 ± 1.0	Pre vs. post: *p* = 0.04 Single vs. double: *p* < 0.001 Time × shift type: *p* = 0.018
Interference score	0.76 ± 0.88	1.38 ± 1.05	0.80 ± 0.78	2.18 ± 1.43	Pre vs. post: *p* = 0.001 Single vs. double: *p* < 0.001 Time × shift type: *p* = 0.001
Response time	32.5 ± 12.5	33.5 ± 9.9	32.1 ± 11.1	41.2 ± 11.1	Pre vs. post: *p* = 0.002 Single vs. double: *p* < 0.001 Time × shift type: *p* < 0.001

The values were presented as mean ± SD, and *p* < 0.05 was accepted as significant.

**FIGURE 2:**
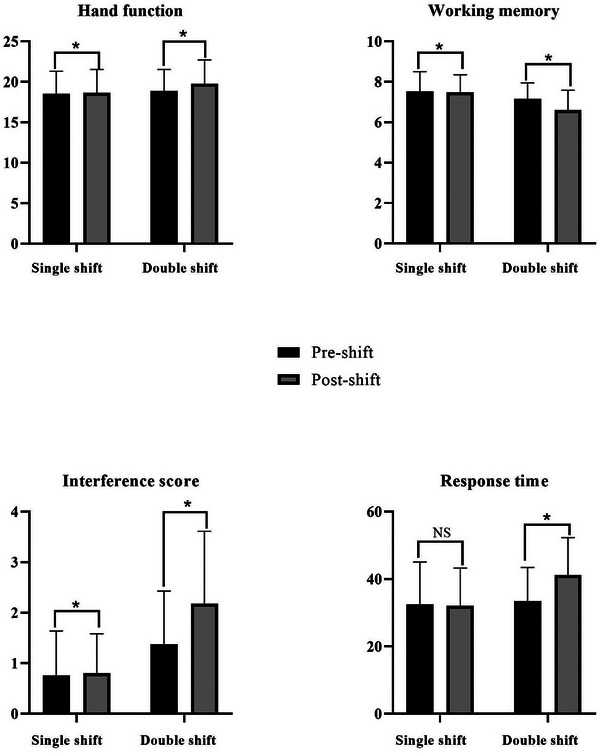
Nurses’ hand and cognitive functions before and after the shifts. (*There is a significant difference (pre‐shift vs. post‐shift), NS: non‐significant.).

Figure 2 displays the nurses' hand function, working memory, interference scores, and response times before and after the shifts. While significant differences were observed in all comparisons, no significant change was noted between pre‐ and post‐single‐shift work in the response time variable. To examine these statistical differences for practical importance, effect sizes (Cohen's d) were computed. The change in hand function (*d* = 0.18) and response time (*d* = 0.09) following the single‐shift work was determined to be practically trivial. The variables of working memory (*d* = 0.33) and interference scores (*d* = 0.64) following the single‐shift work, as well as hand function (*d* = 0.42) values following the double‐shift work, demonstrated small to medium effect size. On the other hand, the values for working memory (*d* = 0.95), interference score (*d* = 1.42), and response time (*d* = 0.82) before and after a double shift exhibited a large effect size.

## DISCUSSION

In healthcare contexts, a nurse's competence is frequently gauged by their ability to deliver high‐quality care to patients (Burston et al., 2014; Kurup et al., 2024). Among a myriad of skill sets integral to nursing, manual dexterity is crucial due to its immediate influence on patient care and safety (Cho et al., 2020). It directly influences the quality and efficacy of patient care, especially in procedures necessitating fine motor control such as the administration of injections or wound suturing (Kuzgun & Denat, 2018). Furthermore, nursing's clinical duties often require the simultaneous execution of multiple tasks, frequently under high‐stress conditions (Singh et al., 2020; Ulupınar & Erden, 2024). This study, therefore, underscores the potential adverse impacts on manual dexterity due to the demanding nature of nursing care, which necessitates a substantial degree of hand–eye coordination and precision. Our study demonstrated a statistically significant decline in nurses' manual functions following both single‐ and double‐shift duties. Upon evaluation of the practical importance of these findings, we discovered that the effect size of a single shift on nurses' hand functions was not significant. Conversely, a double shift had a small, yet appreciable effect on the deterioration of nurses' hand functions.

Working memory is a fundamental cognitive function of paramount importance in the field of nursing, where professionals must continuously update, manipulate, and recall patient information to provide effective and efficient care (Esmaily et al., 2022; Giorgi et al., 2020; Molzof et al., 2019). The complexity and dynamic nature of nursing tasks necessitates strong working memory capabilities (Molzof et al., 2019). As suggested by empirical research, working memory plays an instrumental role in nurses' decision‐making processes, clinical judgment, and execution of procedural tasks (Caruso, 2014; Esmaily et al., 2022; Ganesan et al., 2019). Working memory facilitates the comprehension of patient‐specific information, guides the prioritization of care‐related tasks, and enables the cognitive flexibility necessary for adjusting actions in response to rapidly changing patient conditions. Furthermore, it provides the cognitive resilience to manage and integrate multifaceted streams of information under stressful conditions that typify the nursing environment (Molzof et al., 2019; Pulido‐Martos et al., 2012). Hence, working memory not only underlies cognitive competency in nursing practice but also contributes to the safety and quality of healthcare services (Cho et al., 2020; Dall'Ora et al., 2016). Our results indicated that both single and double shifts significantly changed working memory. However, practically the effect is smaller after a single shift but greater following a double shift. These findings highlighted the cognitive strain that prolonged working hours can impose on nurses, potentially affecting their performance and the quality of patient care.

Attention and response time are critical cognitive factors in nursing, profoundly influencing the efficacy of care delivery in this demanding profession (Esmaily et al., 2022; Ganesan et al., 2019). Attention, the capacity to selectively focus on specific information while ignoring distractors, is crucial in nursing environments, often characterized by a multitude of stimuli and potential interruptions (An et al., 2022; Niu et al., 2013). A nurse's ability to effectively filter and process relevant information directly impacts their ability to prioritize care tasks and respond to emergent patient needs. Concurrently, response time, the interval between stimulus presentation and a nurse's reaction to it, reflects the efficiency of their cognitive processing and decision‐making capabilities (Esmaily et al., 2022; St. Hilaire et al., 2017). Shorter response times are typically associated with swift and effective interventions, directly correlating with improved patient outcomes (An et al., 2022). The findings reveal a substantial reduction in attention following both single and double shifts, while a marked decrease in response time was only observed after a double shift. An important observation from the study is that the decrease in performance parameters following double shifts exhibits a large effect size in practical terms. These findings suggest that extended work hours can considerably affect a nurse's ability to concentrate and respond promptly, critical factors in delivering effective healthcare.

The present findings are consistent with previous research on the impact of shift work on cognitive functions in nurses. For instance, a study by Durán‐Gómez et al. (2021) revealed significant declines in cognitive performance and prefrontal cortex activity among ICU nurses following night shifts (Durán‐Gómez et al., 2021). This decline was linked to a reduction in dorsolateral prefrontal cortex reactivity due to sleep deprivation, resulting in a marked decrease in verbal fluency performance. Similarly, a systematic review by Leso et al. (2021) highlighted that night shifts and long working hours could severely impair cognitive domains such as attention, memory, and response inhibition (Leso et al., 2021). These studies emphasize the negative impact of shift work and prolonged working hours on critical cognitive functions, corroborating our findings. Consequently, our results underscore the need for healthcare institutions to reconsider shift scheduling practices to better protect nurses' cognitive functions and overall performance.

A study conducted on nurses in Taiwan demonstrated that long‐term shift work significantly affects job stress, sleep quality, and overall health. Nurses reported moderate job stress and poor sleep quality, which directly influenced their perceived health status (Lin et al., 2014). While our study did not directly assess job stress or sleep quality, the observed decline in hand function and cognitive performance after double shifts may reflect similar underlying factors such as stress and fatigue associated with extended working hours. Another study investigating the cognitive performance of nurses during different shifts found that working memory and attention significantly decreased after night shifts, while response time was less affected (Esmaily et al., 2022). Our results are consistent with these findings, showing significant impairments in working memory, attention, and response time following double shifts. This indicates that extended shifts, particularly night shifts, can severely impact critical cognitive functions necessary for patient care. While our study did not directly assess job satisfaction, the observed impairments in cognitive and manual functions following extended shifts could potentially contribute to lower job satisfaction, as decreased performance may increase the likelihood of errors and job dissatisfaction among nurses (Moradi et al., 2014).

The research conducted presents substantial evidence regarding the impact of shift work on the hand and cognitive functions of nurses, with a particular focus on the “greater effect” of double‐shift compared to single‐shift. The continuous, sleep‐deprived duration of 16‐h shifts poses a considerable challenge for tasks requiring mental acuity and delicate dexterity. Our findings suggest that such work arrangements should be circumvented whenever possible to maintain the highest level of function and performance in nursing. However, when working conditions are of lower intensity and allow for it, the incorporation of alternating sleep cycles could be considered. For example, integrating periods of sleep, such as a 3‐h rest, within the 16‐h shift could potentially help to alleviate the negative impacts associated with extended working hours.

In shift work scenarios, practices like napping and caffeine consumption are commonly used to combat the increased sleepiness associated with night shifts. However, in Turkey, napping during shift duties is not permitted. Furthermore, for the purpose of this study, we requested that the nurses maintain their regular caffeine consumption habits. This methodology provides a strong basis for believing that the results gathered are generalizable. By maintaining the participant's routine habits and evaluating them in their natural working conditions, we have maximized the real‐world applicability of our findings. This study, therefore, confirms that despite the usage of caffeine, an increase in drowsiness and impairments in cognitive functions were observed towards the end of the double shift, indicating sleep deprivation. These findings underscore the crucial importance of adequate rest and appropriately structured work schedules in maintaining cognitive function and overall performance in the nursing profession. It highlights the need for employers and policymakers to address these factors to ensure the optimal health and productivity of nursing staff.

Despite the insightful findings of our research, it is important to acknowledge that our study has some limitations. The first limitation is that all of our participants were female, which restricts the applicability of our results to the male nursing population. The second limitation was the small sample size, which might restrict the broader applicability of our findings. The third limitation was that our post‐shift testing might have been influenced by factors beyond fatigue, such as time pressure since the participants were eager to leave the workplace. Furthermore, we did not assess the impact of social and family responsibilities on the nurses' cognitive and executive functions. Finally, the menstrual phase of the female participants, which could potentially impact cognitive function and overall performance, was also not considered. On the other hand, one of the major strengths of this study lies in its real‐world setting and the comparative analysis of hand function and cognitive performance across single‐ and double‐shift work settings. These aspects lend practical relevance to our findings, despite the acknowledged limitations.

## CONCLUSION

The evidence from this study underscores the profound impact that extended shift work can have on critical nursing skills, such as precision dexterity, working memory, attention, and response time. The present study, therefore, suggests that there is an urgent need for the development and implementation of strategies to mitigate the deleterious effects associated with prolonged shift work in the nursing sector. Potential strategies could include implementing appropriate rest breaks, allowing for adequate recovery periods between shifts, providing training to enhance resilience and coping mechanisms, and creating a supportive workplace environment. However, further research is needed to comprehensively explore the effects of double shifts and establish the efficacy of the potential interventions.

## IMPLICATIONS FOR NURSING AND HEALTH POLICY

The findings of this study carry substantial implications for clinical practice, particularly in the management of nursing staff schedules. The observed deterioration in hand function, working memory, attention, and response time in nurses working double shifts underscores the need for a critical reevaluation of shift‐scheduling practices within healthcare settings. By incorporating statistical evidence from our results, where a significant increase in cognitive and motor skill impairments was noted (*p* < 0.05), we can advocate for systematic changes to mitigate these adverse effects.

Current shift scheduling varies widely, with some healthcare systems already experimenting with shorter shifts and longer rest periods. Comparing these practices with our findings suggests that adopting similar strategies could substantially benefit nurses' performance and health. In the long term, the enforcement of improved scheduling practices could reduce nurse burnout and turnover, thus enhancing overall healthcare delivery and reducing errors in patient care.

To this end, healthcare administrators should consider designing targeted interventions, such as implementing mandatory rest periods, optimizing shift rotations, and limiting the number of consecutive night shifts. Such policies could not only prevent fatigue‐related errors but also improve patient care quality by ensuring nurses operate at their optimal cognitive and physical capacity. These insights should serve as a foundational element for policymakers and healthcare administrators aiming to devise regulations that support nurse well‐being and promote a sustainable healthcare environment.

## AUTHOR CONTRIBUTIONS

Fadime Ulupınar and Sibel Meler collaboratively designed the study and collected the data. Fadime Ulupınar led the data analysis with contributions from Sibel Meler. The supervision of the study was conducted by Fadime Ulupınar. Both authors, Fadime Ulupınar and Sibel Meler, were involved in the writing of the manuscript. Sibel Meler provided critical revisions for important intellectual content, ensuring the accuracy and integrity of the work.

## CONFLICT OF INTEREST STATEMENT

The authors declare no conflicts of interest related to this research study.

## FUNDING INFORMATION

This research did not receive any specific grant from funding agencies in the public, commercial, or not‐for‐profit sectors.

## ETHICAL CONSIDERATIONS

Ethical approval for this study was granted by the Erzurum Technical University Scientific Research and Publication Ethics Committee, with the Ethics Approval Number 5/9, dated August 17, 2023. Necessary permissions were obtained from the healthcare institutions where the participating nurses are employed. The study was conducted in full compliance with all applicable ethical rules and regulations, ensuring the protection of participant confidentiality and rights. Informed consent was obtained from all participants. The form of consent was written, ensuring that each participant was fully informed of the study's aims, the procedures involved, potential risks, and their rights, including the right to withdraw from the study at any point without any consequences.
